# Machine Learning and Lexicon Approach to Texts Processing in the Detection of Degrees of Toxicity in Online Discussions

**DOI:** 10.3390/s22176468

**Published:** 2022-08-27

**Authors:** Kristína Machová, Marián Mach, Kamil Adamišín

**Affiliations:** Department of Cybernetics and Artificial Intelligence, Faculty of Electrical Engineering and Informatics, Technical University of Košice, Letná 9, 04200 Kosice, Slovakia

**Keywords:** web mining, detection of degrees of toxicity, machine learning, lexicon approach, text data processing

## Abstract

This article focuses on the problem of detecting toxicity in online discussions. Toxicity is currently a serious problem when people are largely influenced by opinions on social networks. We offer a solution based on classification models using machine learning methods to classify short texts on social networks into multiple degrees of toxicity. The classification models used both classic methods of machine learning, such as naïve Bayes and SVM (support vector machine) as well ensemble methods, such as bagging and RF (random forest). The models were created using text data, which we extracted from social networks in the Slovak language. The labelling of our dataset of short texts into multiple classes—the degrees of toxicity—was provided automatically by our method based on the lexicon approach to texts processing. This lexicon method required creating a dictionary of toxic words in the Slovak language, which is another contribution of the work. Finally, an application was created based on the learned machine learning models, which can be used to detect the degree of toxicity of new social network comments as well as for experimentation with various machine learning methods. We achieved the best results using an SVM—average value of accuracy = 0.89 and F1 = 0.79. This model also outperformed the ensemble learning by the RF and Bagging methods; however, the ensemble learning methods achieved better results than the naïve Bayes method.

## 1. Introduction

Social networks today allow us to express publicly our agreement or disagreement with other people, their opinions or social behavior. This freedom is often abused in the online space and that is why we can see social networks that are full of toxic comments dealing with the ongoing pandemic, political or social situations. The increase in textual data on the Internet has stimulated the emergence of new scientific fields that examine short texts in the online space and look for toxic posts, trolls, that try to detect the polarity of comments, and that try to solve other tasks within the processing of textual data.

We focused our research on toxicity detection while distinguishing several degrees of toxicity. Our research was motivated by a serious problem of the spread of toxicity and anti-social behavior in the online space that almost all of us use. All types of antisocial behaviors affect democracy in many countries and contribute to the polarization of a society (Tristan Harris—former expert on the ethics of a design in Google, and cofounder of the Center for Human Technologies). Often, the spreading of toxic posts tries to manipulate the opinions of users looking for answers in the online space. Modern social media companies utilize constant monitoring of users to keep their attention and consequently to ensure the success of advertising. These information technologies can, thus, channel users toward content that causes rudeness, a lack of trust, loneliness and societal polarization, and they can indirectly help electoral manipulation and the spread of populism. For these reasons, we consider it important to help with the automatic monitoring and removal of inappropriate content on social media.

We can divide all toxic posts and comments into types based on the cause of toxicity, for example: cultural–ethnically based, insulting a cultural–ethical group of people; racially based, insulting one race; based on religion; sexually based; physically based—insults that are based on health or physical condition adaptations; personal, insulting a specific person; politically based—insults that are based on a political opinion.

Such online harassment affects both adults and children. Research published in [1] focused on identifying the groups of people who are most at risk. The most influential attributes of online harassment in adults are age and gender and this mostly concerns young men aged 18 to 29 and women aged 18 to 24.

Study [2] analyses hate speech using a web interface with a focus on the most used social networks, such as Twitter, YouTube, and Facebook. Individual social networks try to protect their users. For example, users who are bothered by inappropriate posts can report these posts. Then, the administrators of social networks check the reported posts and delete them if they are really toxic, but this manual regulation is no longer enough today; therefore, automated systems are being developed to detect toxicity. This automatic detection is based on either machine learning methods or a lexicon-based approach.

We have used a lexicon-based approach in the past in our work focused on the detection of suspicious online reviewers [3]. In this work, we used a lexicon-based approach in a sentiment analysis method as an alternative to machine learning methods for the detection of trolls. Here, we instead use a lexicon-based approach in a new method for the automatic labelling of extracted posts in a dataset. We can summarize the contribution of our paper as follows:

- Creation of a dataset by extracting comments from social media in the Slovak language. There is lack of text data for processing in the Slovak language.
- Automatic labelling of extracted data using our lexicon-based method, which represents our original approach to texts labelling.
- Creation of a lexicon of toxic words in the Slovak language as an essential resource needed for the lexicon-based labelling method.
- Comparison of detection models trained by classic machine learning methods (e.g., naïve Bayes, and SVM) and ensemble learning (e.g., Bagging, and RF).
- Creation of an application using the detection models.

### 1.1. The Toxicity Detection Using Machine Learning

The detection of the degrees of toxicity in online discussions is a multi-classification problem; therefore, using the classification methods of supervised machine learning methods is a natural choice. An example of this approach is the work published in [4], which presents an approach to determine the toxicity of vulgar posts on Twitter using partial learning with a teacher and the logical regression method, where they achieved a true positive rate of 75.1%.

Determining hateful comments in the Italian language was dealt with in the work of [5]. They used SVM (support vector machine) and LSTM (long short-term memory) models trained on a sample of 17,000 comments from Facebook. For the experiment, they used only the data that was annotated by at least three annotators. They divided the comments into three classes: comments without hate, comments with a slight hint of hate, and hateful comments. They further divided the hateful comments into categories based on what the comments were about, namely, religion, physical or mental health, socio-economic status, politics, race, or gender. The result was two documents, one with 3356 comments, divided into 2816 non-hateful comments, 410 mildly hateful comments, and 130 strongly hateful comments. The other document had 3575 comments, divided into 2789 non-hateful comments and 786 hateful comments. Their best results were an accuracy of 80.60% using an SVM and 79.81% using a LSTM for the model training.

A work dedicated to the Arabic language [6] detected the use of offensive words. To create the model, they chose data from Aljazeera.net, which contained 400,000 comments on approximately 10,000 articles. Subsequently, 32,000 offensive comments were selected from them. The selected comments were annotated using CrowdFlower, which divided them into three groups: obscene, offensive, and neutral comments. They achieved their best result of a 0.98 precision using the LOR (log odds ratio) with unigrams.

Since online comments or posts are often informal, unstructured, and poorly written, a problem arises when classical models are trained to detect toxicity. Due to these problems, the authors in [7] proposed to detect the toxicity of posts using the lexical syntactic feature (LSF) architecture, which is used to detect offensive content and to identify potential offensive users in interactive media. As a result, the LSF architecture performed significantly better than the existing methods in detecting toxic posts, achieving a 98.24% accuracy in detecting toxic (attacking) posts and up to a 77.9% accuracy in identifying those users. This method, however, was only able to find 78.86% of toxic contributions.

Additionally, the work of [8] used the SVM, RF and naïve Bayes machine learning methods combined with TF-IDF (term frequency—inverse document frequency) and word embedding representations for cyberbullying and aggressiveness detection in Tweets in the Chilean and Mexican Spanish languages. In this work, all the SVM models obtained better results than the others, with up to 89.2% accuracy and an 89% F1 rate.

Moreover, the work of [9] used neural network methods (e.g., BiGRU—bidirectional gated recurrent unit and BiLSTM—bidirectional long short-term memory) and classic methods of machine learning in combination with TF-IDF and GloVe (global vector) representations for cyberbullying detection. Across all the preprocessing steps, the logistic regression displayed the highest average performance amongst all the machine learning techniques used, followed by SVM, XG Boost (extreme gradient boosting), and naïve Bayes in that order. They achieved the best results using neural networks—with accuracy and F1 scores as high as ~95% and ~98%, respectively.

The machine learning approaches are also commonly used for sentiment analysis. Sentiment analysis is, however, related to toxicity recognition, because toxicity in online spaces usually represents a negative opinion. There are some works which have used machine learning approaches for sentiment analysis, for example, [10] developed an ensemble learning scheme using DT (decision trees), SVM, RF (random forest—of decision trees) and KNN (k-nearest neighbors) for a sentiment analysis of COVID-19 related comments. Additionally, in the work of [11], deep learning models for a sentiment analysis were used in recommender systems. There are also some related works using sentiment analysis based on machine learning.

### 1.2. Lexicon-Based Approaches to Toxicity Detection

The lexicon-based approach was originally used for the sentiment analysis of texts, but it has also been used in the creation of recommender systems [11]. The detection of toxicity using a lexicon-based approach analyses individual words, phrases and post or comment sentences using lexicons. There are several types of lexicons and they can be divided according to the language they use (the most widespread are English lexicons) and the goal of analysis on what words are used (for example, the recognition of toxic, positive, or negative posts). Lexicons can also be created by merging several other lexicons, translating foreign language lexicons into another language or by adding certain words to the already existing lexicon that can help us in recognition toxicity.

WordNet is the most famous lexicon, which contains a database of English words such as nouns, adjectives, adverbs, and verbs, that are grouped into synonym sets or so-called synsets. WordNet contains 117,000 English synsets and groups them according to their meaning. WordNet also indicates the semantic relationships between words [12].

The Macquarie Semantic Orientation Lexicon (MSOL) is an English dictionary that contains 30,458 positive and 45,942 negative words and phrases. It is a generated lexicon created using a Roget-like thesaurus and Macquarie thesaurus [13].

A very important issue in lexicon building is the correct annotation of words in the lexicon by a measure of toxicity. This annotation can be provided by humans, but more efficient is an annotation using an optimization method. In the paper of [14], PSO (particle swarm optimization) and BBPSO (bare-bones particle swarm optimization) were used. The problem with lexicon-based approaches is that they cannot extract opinions with a specific orientation. In that work, the corpus-based approach was used to solve this problem. For universal words that are not specific to the domain, they look for other sentimental words in a corpus that already have their specific orientation and then adapt this universal lexicon for a new list of sentimental words specific to the given corpus.

## 2. Materials and Methods

When solving the task of detection, namely, the degree of toxicity of online posts, we decided to use a hybrid method, which consisted of two approaches in two steps. The lexicon-based approach was used in the first step to label the toxic comments in the dataset. The machine learning approach was then used in the second step to train the classification models using machine learning methods.

For training the models, we used our own text data, which we labelled with the degree of toxicity using the lexicon approach. The data was extracted from Facebook and Instagram in the Slovak language. From the machine learning methods, we decided to use classical methods such as naive Bayes, random forest, SVM and bagging, which are usually successful in text classification.

### 2.1. Data Description

We prepared the dataset by downloading the comments in the Slovak language from Facebook and slightly fewer comments from Instagram. The posts that contained comments came primarily from the news profiles, namely, “*Televízne noviny TV Markíza*” and “*Television TA3*”. Several dozen comments also came from the public profiles of specific people. We downloaded the comments in two ways. The first was an extraction using the freely available Export Comments downloader (http://exportcomments.com/ (accessed on 26 July 2022)). To download using this tool, it is necessary to enter the URL address of the post as an input, and then download them in .xlsx or .csv formats. This method of data extraction has its limitations.

The second method we used was to create our own tool for the comment extraction. This was implemented in the Python language, with help from logging into our Facebook account and sending a POST request to “https://m.facebook.com/login.php” (accessed on 26 July 2022) with data stored in the JSON format that contained our login information. After logging into the account, we gave the program the URL address of the post on Facebook that we wanted to download. Subsequently, the page data was retrieved using a GET request. Each comment on the page was stored in the same *<div>* variable and marked with the same class. After searching for comments in the code, we saved them in a .csv file.

The comments downloaded by us had to be pre-processed before further use. The Slovak language is characteristic in its use of common diacritical marks. It is the same with punctuation marks—periods, commas, question marks, exclamation points, colons, and others. Some people on social networks use diacritical marks and punctuation marks, but most people do not use them. When processing the comments, it was necessary to unify the texts and remove all of them. Another pre-processing step was to convert uppercase letters to lowercase letters, since our lexicon only contains words with lowercase letters. The uppercase letters when determining toxicities play almost no role. Some comments also contained an image or a link to another page, which were also unusable for our model training, and deleted.

The resulting dataset contained 3092 labelled texts of posts. This dataset titled, “Toxic_training_data.csv” is available at: https://kristina.machova.website.tuke.sk/ (accessed on 26 July 2022), in the folder RESEARCH at the end of page between “Useful links” (https://kristina.machova.website.tuke.sk/useful/Toxic_training_data.csv (accessed on 26 July 2022)).

### 2.2. Used Lexicon Approach for Data Labelling

The lexicon-based approach was used in our work to find toxic words from a pre-created lexicon in the extracted comments, and then to label these comments with their degree of toxicity. This labelling is necessary so that we can train detection models to determine the degree of toxicity of unknown comments using supervised learning methods. We classified the comments into four classes, namely:

- *neutral posts*—do not contain any negative words from the lexicon marked by us,
- *weakly toxic posts*—contain fewer toxic words from the lexicon,
- *moderately toxic posts*—contain words from the group of moderately toxic words from the lexicon,
- *very toxic posts*—contain extremely toxic words of the lexicon.

We have created the lexicon for labelling using our domain-independent dictionary created in the past, “lexicon_Small_human.json” (available at: https://kristina.machova.website.tuke.sk/ (accessed on 26 July 2022) in the folder RESEARCH at the end of page between “Useful links”) for determining the polarity of posts, which contained negative words (also positive, but we did not take them into account in this work). We edited and supplemented this lexicon of negative words with toxic words of varying degrees of toxicity. We annotated the degrees of the toxicity of words manually. In the lexicon, there are words labelled with the values “1” for the least toxic words, “2” for moderately toxic words and “3” for very toxic offensive words and swear words in the Slovak language. The dictionary is stored in the JSON format and contains a total of 809 toxic and offensive words, of which there are:

- 224 words with a toxicity level “1” (mildly toxic words),
- 243 words with a toxicity level “2” (moderately toxic words),
- 342 words with a toxicity level “3” (very toxic words).

The resulting Slovak language lexicon “Lexicon_of_toxic_words.json” is available at: https://kristina.machova.website.tuke.sk/useful/Lexicon_of_toxic_words.json (accessed on 26 July 2022).

For the pre-processing of data sets, the lexicon-based approach to labelling and use of training methods, we used the Java programming language. After pre-processing, each comment was divided into an array of words—tokens—and stored in a variable. The evaluation of the comment was carried out from individual words in this field, where each word was looked up in the created toxic lexicon and the toxicity value of the word was returned. We used two ways to evaluate the final toxicity of a comment:

1. the label value is the sum of the individual toxic words in the comment,
2. the label value is the value of the most toxic word in the comment.

When learning and testing the models, we found that the second method of determining the final value of a comment as the value of the most toxic word achieves better results. A comment that did not contain any words from the toxic lexicon was labelled as neutral. The values of the labels are in the form of a “float”, and we needed to create some differences between the degrees of toxicity of the comments. In our case, they were set as follows:

- the value 0.0 belongs to Neutral,
- the value 1.0 belongs to Low toxic,
- the value 2.0 belongs to Moderately toxic,
- values 3.0 and more belong to Very toxic comments.

**Figure 1:**
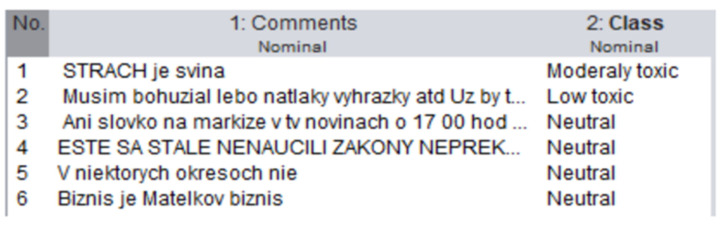
Illustration of the dataset of labelled comments extracted from social networks.

The dataset of labelled comments using the lexicon-based approach was subsequently stored in the form of a CSV file with the columns “Comments” (containing comments in the form of text (string)) and “Class” (containing the values Neutral, Low toxic, Moderately toxic or Very toxic in the form of a nominal attribute). An illustration of the data can be seen in Figure 1. To use the Weka tool for model training, we needed to convert the data from CSV to ARFF.

### 2.3. Used Methods for Models Training

We used classical learning as well as ensemble learning for training the detection models. From the classic machine learning approaches, we chose the naïve Bayes classifier as a baseline method, as well as an SVM, because of its performance in related works [5,8,9,10]. This method is very efficient in text data processing. From the ensemble methods, we selected bagging as a basic approach and random forests, because it could increase the precision of the final classification by vote among a set of de-correlated tree models.

The naïve Bayes classifier (NB) is a probabilistic classifier based on Bayes’ theorem and independence assumption between features. The advantage of this algorithm is its simplicity and clarity, as each class is characterized by a probabilistic description. On the contrary, the disadvantage of the method, as mentioned, is the assumption that attributes are independent. NB is often applied as a baseline method; however, it is reported to be outperformed by support vector machines [15].

**Figure 2:**
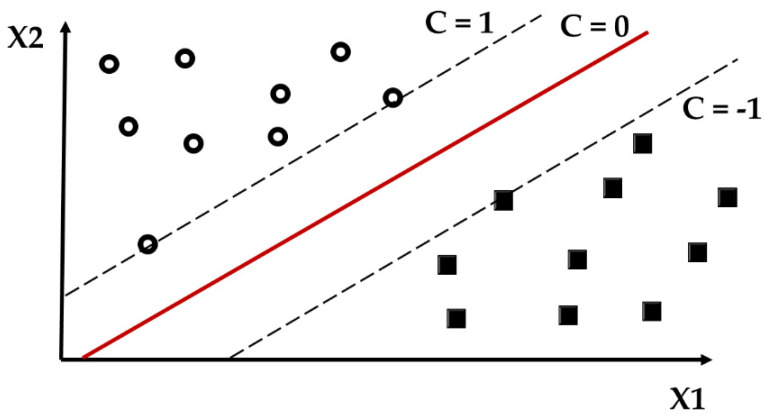
Illustration of the linear SVM.

Support vector machines (SVM) separate the sample space into two or more classes with the widest margin possible. The method was originally a linear classifier, which creates a decision boundary for the separation of examples in the space into two classes “1” and “−1” [16], as can be seen in Figure 2.

**Figure 3:**
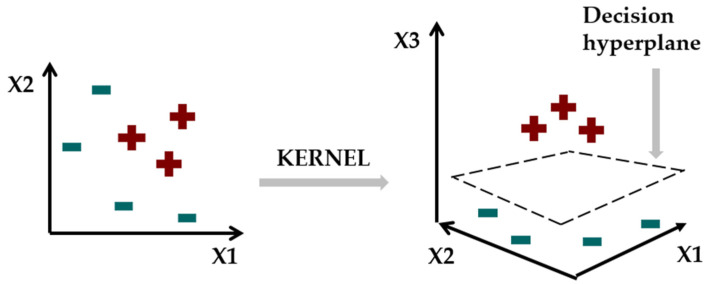
Illustration of transformation of the n-dimensional space into n + 1 dimensional space using a kernel function (there are examples of two classes – the first one consist of red pluses and the second one consist of green minuses).

The linear SVM is suitable only for linear data, therefore, it is not a good selection for text data, but it can relatively efficiently perform a non-linear classification using kernel functions [17]. The trick of kernels functions is illustrated by Figure 3.

The objective is to maximize the width of the boundary, which is known as the primal problem of support vector machines [18].

**Figure 4:**
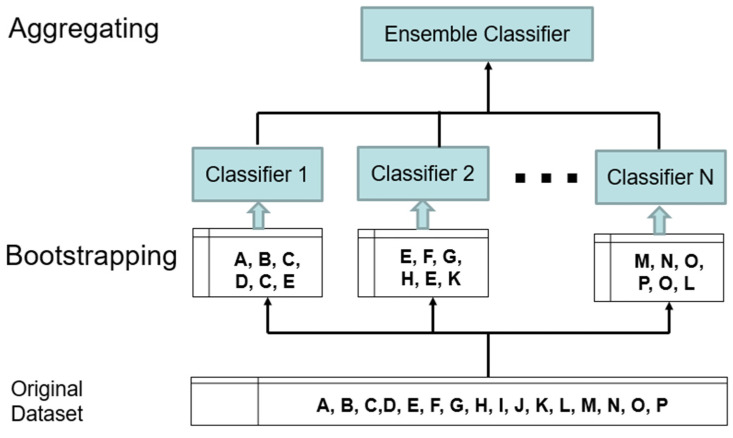
Illustration of two steps of Bagging method—Bootstrapping and Aggregating.

Bagging is a classification method belonging to the group of learning by a set of methods—so called ensemble learning—which helps improve the stability and accuracy of machine learning. The algorithm creates several Bootstrap samples so that each sample works as an independent dataset. After selecting “*m*” samples, a partial classifier is generated for each of them. Then, the results are averaged, and the resulting class is selected by the vote of all partial classifiers [19], which is illustrated in Figure 4.

The random forests (RF) method also belongs to the ensemble learning group of methods. It is based on decision trees (DT) and the number of DTs is a parameter. The random forests method tries to minimize the variance by creating de-correlated DTs using a random selection of a subset of attributes. The method determines the result by a vote of the individual generated trees [20].

The complexities of the used and proposed methods are different. The naïve Bayes algorithm has the complexity *O(N*×*M)*, where *N* is the number of attributes – words and *M* is the number of examples in the dataset. The complexity of the SVM depends on the used optimization. Using quadratic programming, the complexity becomes *O(N^3^)*, but the complexity of the sequential minimal optimization depends only on support vectors. The complexity of decision trees is *O(N*×*M)* but for RF it is *O(K*×*N*×*M)*, where *K* is the number of generated trees. Bagging also has a similar complexity. The complexity of our lexicon-based labelling method is *O(M*×*L*×*T)*, where *M* is the number of labelled texts in the dataset, *L* is the number of words in the lexicon and T is the number of terms in a unique labelled text. 

## 3. Methodology of Research

**Figure 5:**
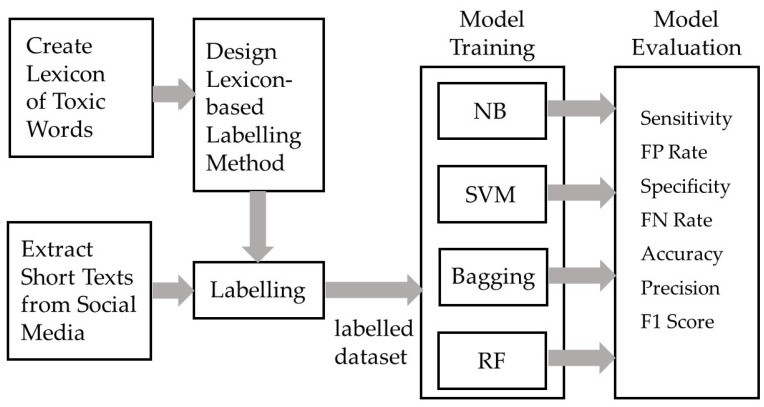
Methodology of research devoted to the creation of the detection models for detection of a degree of toxicity in social media.

Our research was focused at first on the creation of a new lexicon of toxic words in the Slovak language, which was an inevitable resource for our lexicon-based labelling method. The resulting Slovak language lexicon, “Lexicon_of_toxic_words.json” is available at: https://kristina.machova.website.tuke.sk/useful/Lexicon_of_toxic_words.json (accessed on 26 July 2022). The lexicon was created from English lexicons by the translation of negative words to the Slovak language and it was extended by toxic words in the Slovak language. Next, we designed a lexicon-based labelling method. We extracted short texts from social media to create a dataset, which was then labelled using our lexicon-based method. The labelled dataset (available at: https://kristina.machova.website.tuke.sk/useful/Toxic_training_data.csv (accessed on 26 July 2022)) was used for the training of the classic methods (NB, SVM) and ensemble methods (bagging, RF). The models were evaluated using the sensitivity, FP rate, specificity, FN Rate, accuracy, precision and F1 score. Then, the best models were used in the application for toxicity detection. The methodology is illustrated in Figure 5.

We decided to use the freely available Weka tool to train the classification models. This tool can be used in two ways. The first is to import this tool in Java as a library. With its help, it is possible to use all the functions that the tool contains. The second way is to use the graphical interface of the Weka program. We decided to combine these two methods by using the graphical interface of the Weka program to create, test and save the models, and then, to use the created models for a new data classification, we used the library in the Java language. The advantage of such a combination is the ease of setting parameters when training models using a graphical interface and a simple evaluation of model quality using the Java library.

For the model training, the Weka GUI Chooser offers several tools to choose from. We worked with the Explorer tool. The training consisted of several steps:

- selection of training data,
- use of filters for data pre-processing,
- selection of the required model,
- setting of the model,
- selection of the method of testing the model,
- evaluation of the achieved results.

In the process of the model training, we started from the parameters that were predefined in the Weka tool and then changed them until we obtained the most effective model. Manual tuning of the parameters was used. The parameter settings of the best models for each machine learning method are listed in tables below.

The input for training the models was data stored in a CSV file, which contained two columns of data, namely, text data (String) containing comments, and a column with nominal values (Nominal), containing the toxicity value of the given comment. The total number of 3091 comments were divided into following categories:

- Moderately toxic—776 data,
- Low toxic—757 data,
- Neutral—779 data,
- Very toxic—779 data.

**Figure 6:**
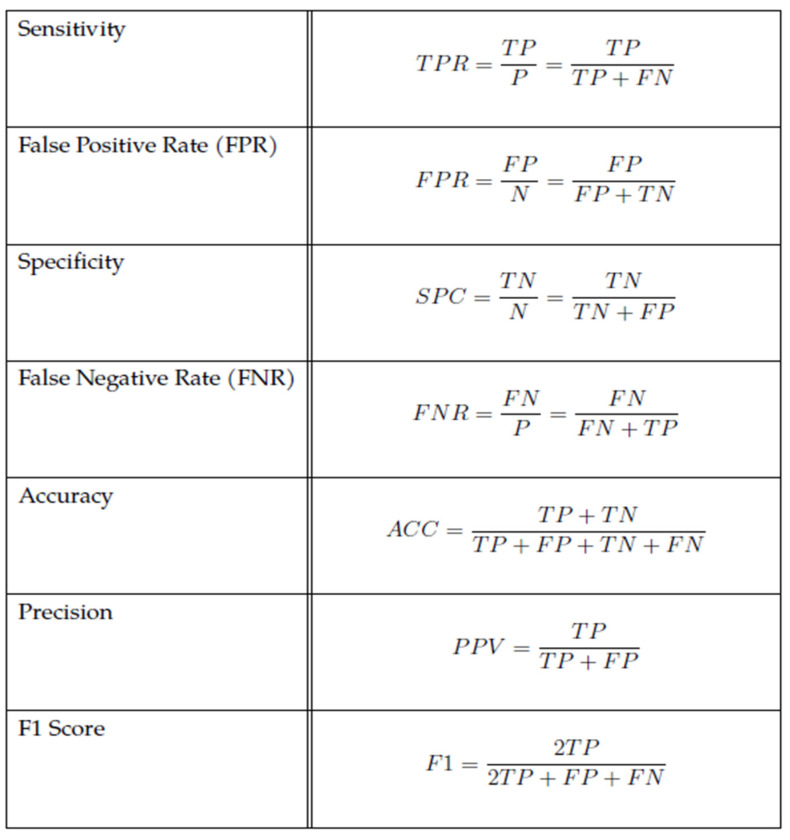
Formulas of the used measures of the effectivity of models.

We used the following measures of effectivity of the models: Sensitivity, False Positive Rate, Specificity, False Negative Rate, Accuracy, Precision and F1 Score. They were calculated according to the formulas in Figure 6 where:

- TP is the number of true positive classifications,
- FP is the number of false positive classifications,
- TN is the number of true negative classifications,
- FN is the number of false negative classifications.

### 3.1. Models Training Using Classic Methods of Machine Learning

We have focused at first on classic methods such as naïve Bayes multinomial (NBM) and support vector machines (SVM). These methods generate models that can be intuitively well explained. Naïve Bayes offers conditional probabilities, which represent the measure of belonging attributes to classes for recognition. Support vector machines are often used because they form a mathematical model of a hyperplane dividing two or more classes in a space. The model contains information about the importance of particular attributes in it. The baseline method for the experiments was the NBM.

With the naïve Bayes method, there are several ways we can create models. The first one is to build a model using the function “*NaiveBayesMultinomial-Text*”. In this case, the “*FilteredClassifier*” function is not used, but the data is processed directly without a filtered classifier. The training inputs are full sentences from the comments along with the labelling obtained using the lexicon approach. The settings which we achieved the best results with are listed in Table 1. The NBM—Text model was validated using a 10-fold cross-validation. This means, that 9/10 of the data were used for training a model, which was consequently validated on an unseen 1/10 of the data, which were not used for training. The total number of data entries was 3091, of which 2086 were correctly categorized, which represents 67.49%, while 1005 data were categorized incorrectly, which represents 32.51%. Detailed validation results are shown in Table 2. These results are not bad if we consider that the random selection for four classes has a precision of 0.25.

The second way to train a naïve Bayes classifier is using the function “*NaiveBayesMultinomial*” in combination with the “*FilteredClassifier*” function. In this case, text input is transformed into the vectors of words. We used “*StringToWordVector*” as a filter. The settings with which we achieved the best results with this model are listed in Table 3 and the validation results are shown in Table 4.

The total number of records during the model training was 3091, of which 1890 were correctly categorized, which represents 61.15%, while 1201 data were incorrectly categorized, which represents 38.85%. The data was validated using a 10-fold cross-validation.

Both naïve Bayes models achieved the best results in specificity and accuracy. The naïve Bayes multinomial text was better in the detection of three degrees of toxicity—very, moderately and low toxic. The neutral comments were better classified by the naïve Bayes multinomial using a vector representation of the text.

The second classic method of machine learning—SVM—was used for training the other models. This method is usually very successful in text processing. We trained two SVM models. For the first one, the function *FilteredClassifier* was used with the filter *StringToWordVector*, which transforms the text of comments into the vectors of words. The settings of training and the filter settings for the SVM1 model are presented Table 5. The validation results are shown in Table 6.

The total number of data entries during the training was 3091 comments, of which 2139 were correctly classified, representing 67.1%, while 952 were incorrectly classified, representing 33.9%. We obtained this result by a 10-fold cross-validation.

The next SVM2 model, with a slightly better result, differed from the previous SVM1 model only in the settings of the filter. Namely, the stemmer attribute had the value *LovinsStemmer*. All other filter and model settings remained the same as for the SVM1. In the following Table 7, we can see an improvement of the results of the SVM2 model in all indicators compared to the previous SVM1 model. The total number of records was 3091, of which there were 2473 correctly classified comments into all classes, which represents 80%, while a total of 618 were incorrectly classified data, which represents 20%. The model was evaluated using a 10-fold cross-validation.

In comparison with the baseline NBM models, the SVM models achieved better results. In addition, a small change in the settings (stemmer = LovinsStemmer) produced better results in the specificity above 90 percent.

### 3.2. Models Training Using Ensemble Learning

In several works, ensemble learning provided better and more reliable results for classification than single machine learning methods. We decided at first to use a basic method of ensemble learning, namely, bagging. Using the bagging method, we generated a set of models. Similar to the previous methods, we used the *FilteredClassifier* function, where we applied a filter with the following settings presented in Table 8. The test results of this model are presented in Table 9.

The bagging method was validated using a 10-fold cross-validation. The total number of training data was 3091, of which there were correctly classified 2312 comments, which represents 74.80%, while 779 comments were incorrectly classified, which represents 25.2%.

The results presented in Table 9 did not confirm the tendency of composite classification by an ensemble of models to give better results than models learned by single machine learning methods. The results of the bagging method were worse than the SVM2 model, although better than the SVM1 model; therefore, we trained and tested one more model using the ensemble learning, namely, a random forest (RF) of decision trees, which is the newest and most successful from the ensemble methods. The “*FilteredClassifier*” function was also used for training the RF. In this function, the RF method was specified as the classifier, and “*StringToWordVector*” was set as the filter. The “*LovinsStemmer*” function was set for the stemmer attribute. We can see the random forest setting in Table 10. The test results of this model are presented in Table 11. They are unfortunately not better than the results of the bagging method.

The RF model was validated using a 10-fold cross-validation. The total number of comments was also 3091, of which 2173 were correctly classified, which represents 70.3%, while 918 comments were incorrectly classified, which is 29.7%. This model achieved the best results among all the models we trained using the random forest method.

### 3.3. Application for Recognition of Degrees of Toxicity

We used the most precise models for recognition of the degrees of toxicity of online comments in the application for analyzing newly extracted posts (they were not used for the model training). For an easier analysis of new data with the option of selecting the type of model, we created an application in Java that includes an annotation using the lexicon-based method, and also allows the use of all the best models. The user can choose which model they want to use to analyze new comments. After starting the program, the first thing that appears is a window with a menu that offers us a choice of several options:

- vocabulary approach—a method based on the lexicon for the searching and annotation of toxic words,
- SVM model—the use of the SVM model to analyze the toxicity of new comments,
- random forest model—analysis of online comments using the random forest model,
- naive Bayes model—using the naive Bayes model to analyze online comments,
- bagging model—analysis using the bagging model,
- info—contains final results for comparing the quality of models,
- exit—ending the program.

**Figure 7:**
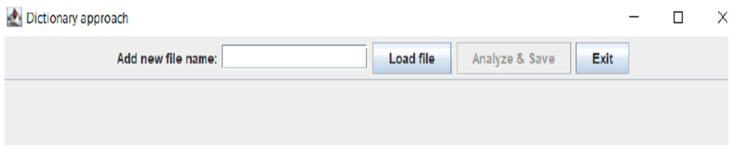
Window for a text data loading and an analysis starting.

After selecting one of these options, a new window will open (see Figure 7), which is used to analyze the text of a comment. In the upper part, we can select the file which contains the text to be analyzed. The program allows us to choose one of two file types, namely, csv or arff.

**Figure 8:**
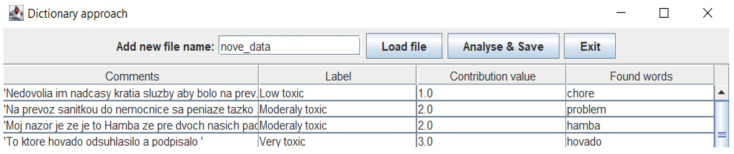
The illustration of results of the lexicon-based analysis of comments from the selected and loaded file.

Next, the user can start the analysis of the selected document of comments and save the results. The analysis produces a table containing the analyzed comments from the selected file together with their toxicity values, as illustrated in Figure 8. A file with this data will also be saved as a csv.

## 4. Discussion and Conclusions

**Figure 9:**
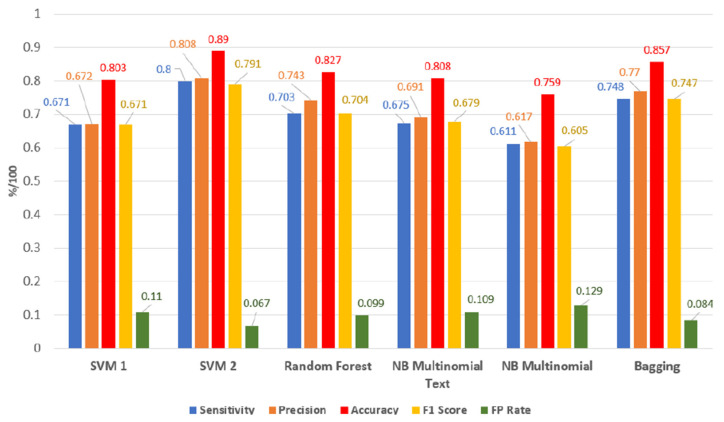
The average results of all models in Sensitivity, Precision, Accuracy, F1 Score and FP Rate measures.

The most accurate model for the degree of toxicity recognition of online comments is the SVM 2 with an average accuracy value of 0.890, which exceeded all other models at each efficiency measure. The second most accurate model is the model created using ensemble learning bagging with an average accuracy value of 0.857, which is only slightly less than the SVM 2. The third most accurate model is the random forest model with an average accuracy value of 0.827. The SVM 1 and naive Bayes multinomial text models have very comparable results with differences in accuracy up to the third decimal place. The least successful is the naive Bayes multinomial model with an average accuracy of 0.759. The average results of all the models are illustrated in Figure 9. Our experiments did not confirm the tendency of composite classification by an ensemble of models to give better results than models learned by single machine learning methods.

Table 12 shows a comparison of the predictive performance of our best model to the best models from previous studies focused on the same or similar problem. We can see from this table of best results, that our SVM model achieved better results than the SVM models of previous studies, but the deep neural network model based on BiLSTM was more effective than our best model. On the other hand, the effectiveness of different approaches as presented in Table 12 can be understood as indicative only. The reason for this is that the experiments, the results of which are collected in the table, were performed by different authors employing different textual datasets (with datasets differing not only in content but in their used language as well). Nevertheless, the table identifies the BiLSTM method as a possible candidate for the next research on detecting toxicity in online discussions in the Slovak language.

This article dealt with the detection of the degree of toxicity of posts in online discussions on social networks. It describes the procedure for training models using both single and ensemble machine learning methods and their comparison. The article also deals with the creation of a lexicon of toxic words and the use of this lexicon to label short texts in a dataset by the degree of toxicity of texts. We can say that the novelty of the article is mainly based on the lexicon approach for the Slovak language and in the comparison of the effectivity of the detection models trained using classic and ensemble learning. An application for the extraction of comments from social media was designed to help with acquiring the text data for the generation of the detection models. We have used the extracted and labelled comments as a dataset to train the classification models. Additionally, an application was created to detect the degree of toxicity in new comments, based on the most successful models.

In future, we could focus on the neural ensemble models [21], since the ensemble models based on classical machine learning methods did not achieve the results we hoped for. In particular, an ensemble could use methods, such as BiLSTM and the more accurate BiGRU according to [22]; we could also extend the text processing to include the processing of images available in online comments, for example, emoticons. Future research could also focus on using various ensemble strategies [23] to increase the detection performance of a set of models, for example, by using the random forest voting strategy.

Another possibility for future research is using new optimization methods in the SVM method, such as ant colony optimization or particle swarm optimization [24], the dynamic feature weight selection [25], and feature extraction integrating principal component analysis and local binary patterns [26]. Using a kernel extreme learning machine could also bring improved results [26].

## Figures and Tables

**Table 1:** The settings of the Naïve Bayes Multinomial Text model training.

Settings	Values
lowercaseTokens	True
minWordFrequency	2.0
norm	2.0
numDecimalPlaces	3
stemmer	LovinsStemmer

**Table 2:** Effectivity of Naïve Bayes Multinomial Text model for recognition of four degrees of toxicity in online discussions.

Measures	Very Toxic	Moderately Toxic	Low Toxic	Neutral
Sensitivity	0.671	0.663	0.742	0.655
FP Rate	0.078	0.061	0.094	0.200
Specificity	**0.896**	**0.918**	**0.874**	**0.773**
FN Rate	0.329	0.367	0.258	0.345
Accuracy	**0.827**	**0.830**	**0.834**	**0.741**
Precision	0.743	0.776	0.719	0.525
F1 Score	0.705	0.697	0.730	0.583

**Table 3:** The settings of the Naïve Bayes Multinomial model training.

Settings	Values
IDFTransform	True
TFTransform	True
lowCaseToken	True
stemmer	SnowballStemmer
wordsToKeep	2000

**Table 4:** Effectivity of Naïve Bayes Multinomial model for recognition of four degrees of toxicity in online discussions.

Measures	Very Toxic	Moderately Toxic	Low Toxic	Neutral
Sensitivity	0.702	0.693	0.651	0.401
FP Rate	0.135	0.172	0.139	0.072
Specificity	**0.812**	**0.773**	**0.812**	**0.904**
FN Rate	0.298	0.307	0.349	0.600
Accuracy	**0.777**	**0.748**	**0.763**	**0.749**
Precision	0.637	0.575	0.603	0.651
F1 Score	0.668	0.629	0.626	0.496

**Table 5:** The settings of the SVM1 model training.

Settings	Values
IDFTransform	True
TFTransform	True
Debug	True
loweCaseTokens	True
stemmer	NullStemmer
wordsToKeep	3000
batchSize	200
kernel	PolyKernel
numDecimalPlaces	3

**Table 6:** Effectivity of SVM1 model for recognition of four degrees of toxicity in online discussions.

Measures	Very Toxic	Moderately Toxic	Low Toxic	Neutral
Sensitivity	0.628	0.723	0.712	0.621
FP Rate	0.093	0.112	0.102	0.132
Specificity	**0.880**	**0.854**	**0.866**	**0.839**
FN Rate	0.372	0.277	0.288	0.379
Accuracy	**0.804**	**0.814**	**0.820**	**0.775**
Precision	0.694	0.684	0.695	0.613
F1 Score	0.659	0.703	0.703	0.617

**Table 7:** Effectivity of SVM2 model for recognition of four degrees of toxicity in online discussions.

Measures	Very Toxic	Moderately Toxic	Low Toxic	Neutral
Sensitivity	0.711	0.791	0.828	0.870
FP Rate	0.044	0.053	0.051	0.119
Specificity	**0.950**	**0.938**	**0.939**	**0.867**
FN Rate	0.289	0.209	0.172	0.128
Accuracy	**0.884**	**0.897**	**0.908**	**0.868**
Precision	0.846	0.834	0.839	0.714
F1 Score	0.773	0.812	0.834	0.785

**Table 8:** The settings of filters in the ensemble learning using Bagging method.

Settings	Values
IDFTransform	True
TFTransform	True
lowCaseToken	True
stemmer	LovinsStemmer
wordsToKeep	1000

**Table 9:** Effectivity of Bagging method for recognition of four degrees of toxicity in online discussions.

Measures	Very Toxic	Moderately Toxic	Low Toxic	Neutral
Sensitivity	0.574	0.719	0.799	0.901
FP Rate	0.052	0.055	0.052	0.184
Specificity	**0.948**	**0.932**	**0.933**	**0.791**
FN Rate	0.426	0.281	0.201	0.378
Accuracy	**0.842**	**0.870**	**0.894**	**0.821**
Precision	0.813	0.813	0.832	0.622
F1 Score	0.673	0.763	0.815	0.736

**Table 10:** The settings of the ensemble learning using Random Forest method.

Settings	Values
breakTiesRandomly	True
debug	True
numDecimalPlaces	3
numIterations	200
seed	10

**Table 11:** Effectivity of Random Forest model for recognition of four degrees of toxicity in online discussions.

Measures	Very Toxic	Moderately Toxic	Low Toxic	Neutral
Sensitivity	0.525	0.713	0.699	0.875
FP Rate	0.031	0.059	0.070	0.237
Specificity	**0.961**	**0.923**	**0.910**	**0.732**
FN Rate	0.475	0.287	0.301	0.125
Accuracy	**0.831**	**0.858**	**0.848**	**0.771**
Precision	0.850	0.803	0.765	0.555
F1 Score	0.650	0.755	0.730	0.680

**Table 12:** Comparison of results in Accuracy of our best model to the best models from previous studies focused on the same respectively similar problem.

Studies	Methods	Type of Detection	Best Results
[5]	SVM, LSTM	Hate posts	Acc (SVM) = 0.699
[7]	LSF *	Offensive posts	Acc (LSF) = 0.778
[8]	SVM, NB, RF	Aggressive language	Acc (SVM) = 0.892
[9]	SVM, LR **, BiGRU, BiLSTM	Cyberbullying	Acc (BiLSTM) = 0.948
Our	NB, SVM, Bagging, RF	Toxic posts	Acc (SVM) = 0.908

* Lexical Syntactic Features, ** Logistic Regression, Acc—Accuracy.

## Data Availability

Machine learning models were trained on the dataset “Toxic_training_data.csv” available at: https://kristina.machova.website.tuke.sk/useful/Toxic_training_data.csv (accessed on 26 July 2022). The lexicon of the Slovak language “Lexicon_of_toxic_words.json” is also available at: https://kristina.machova.website.tuke.sk/useful/Lexicon_of_toxic_words.json (accessed on 26 July 2022).
